# Macular hole and submacular hemorrhage secondary to retinal arterial macroaneurysm – successfully treated with a novel surgical technique

**DOI:** 10.3205/oc000158

**Published:** 2020-08-06

**Authors:** Ramin Nourinia, Nazanin Behnaz, Hossein Hassanpour, Zahra Karjoo, Kiana Hassanpour

**Affiliations:** 1 Ophthalmic Research Center, Research Institute for Ophthalmology and Vision Science, Shahid Beheshti University of Medical Sciences, Tehran, Iran; 2 Department of Ophthalmology, Imam Hossein Hospital, School of Medicine, Shahid Beheshti University of Medical Sciences, Tehran, Iran

**Keywords:** submacular hemorrhage, macular hole, recombinant tissue plasminogen activator, pars plana vitrectomy

## Abstract

**Purpose:** To present a 65-year-old patient with macular hole (MH) and submacular hemorrhage (SMH) secondary to a ruptured retinal arterial macroaneurysm (RAM) which was successfully treated with a novel surgical technique.

**Patient:** A 65-year-old woman presented with a 1-week history of sudden-onset visual loss in her right eye. Her best-corrected visual acuity (BCVA) was hand motion in the right eye. Her fundus examination revealed intraretinal and massive subretinal hemorrhage with macular involvement. An MH with an approximate diameter of 600 microns was also noted. The patient was treated by a standard pars plana vitrectomy (PPV) and internal limiting membrane (ILM) peeling, followed by 10 µg recombinant tissue plasminogen activator injection into the submacular space via the MH with a 25-gauge vitrectomy probe. BCVA of the patient increased to 20/320 and 20/60, one and four weeks after surgery, respectively. Optical coherence tomography (OCT) images confirmed the complete anatomical closure of the MH and the restoration of the outer retinal layers such as the external limiting membrane and the ellipsoid zone.

**Conclusion:** This case report expands our knowledge about the management of MH in combination with SMH after a ruptured RAM. We suggest the use of a vitrectomy probe and MH for subretinal recombinant tissue plasminogen activator (rtPA) injection.

## Introduction

Retinal arterial macroaneurysm (RAM), an acquired focal dilation of a retinal artery, usually occurs in the first three orders of the retinal arterial tree and is typically seen in the elderly with systemic hypertension. Despite the favorable prognosis in most RAM patients, multilevel retinal hemorrhages caused by a ruptured RAM can cause serious visual loss, especially when the macula is involved. Many studies reported that ruptured RAMs with submacular hemorrhage (SMH) have the worst visual prognosis compared with RAMs presenting with other signs, including premacular or vitreous hemorrhages and macular edema [1], [2].

Various mechanisms have been proposed to be the cause of irreversible visual impairment after SMH, including the direct chemical toxicity of iron, hemosiderin, or fibrin for the photoreceptors, blocking the photoreceptors’ oxygenation and nutrient supply by thick subretinal hemorrhage, and finally the contraction of a blood clot which causes mechanical damage to the photoreceptors [3], [4]. A further rare cause of visual loss after ruptured RAMs is macular hole (MH) formation. In a retrospective analysis of 75 eyes with RAMs, Tashimo et al. reported that MH occurred in 5.2% of the patients [5].

Although some studies reported the presence of MH in combination with SMH following a ruptured RAM, successfully treated cases have been demonstrated in limited studies [6], [7]. In this case report, we present a 65-year-old patient with MH and SMH secondary to a ruptured RAM, which was successfully treated with a novel surgical technique.

## Case description

**Figure 1:**
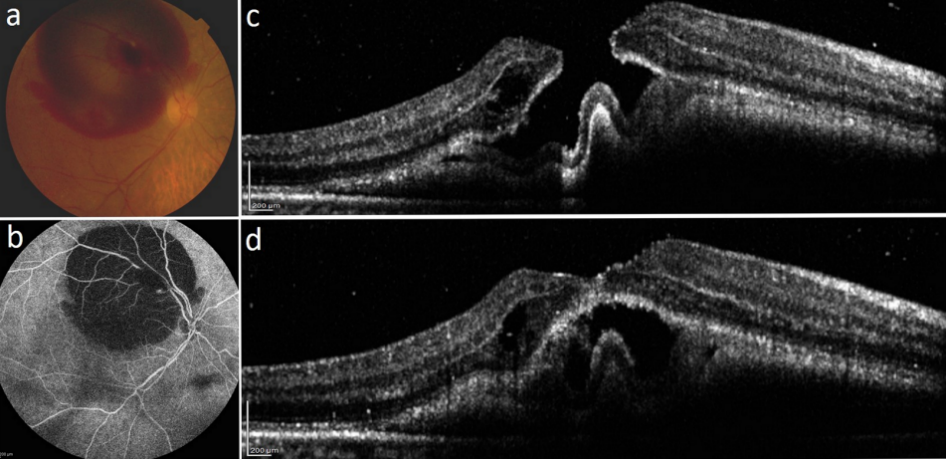
a) The clinical photograph of the right eye demonstrates the presence of a full-thickness macular hole and massive multilevel retinal hemorrhage secondary to a ruptured retinal arterial macroaneurysm in the superotemporal arcade area. b) The fluorescein angiography study confirms the presence of a retinal arterial macroaneurysm in the superior temporal arcade. c) and d) show optical coherence tomography scans with 3-D reconstruction confirming the macular hole 600 µm in diameter.

A 65-year-old woman presented to our clinic with a 1-week history of sudden-onset visual loss in her right eye. She had poorly controlled systemic hypertension. She was otherwise healthy and had no prior ocular history except for mild nuclear sclerosis in both eyes. At the presentation time, her best-corrected visual acuity (BCVA) was hand motion in the right eye. The relative afferent pupillary defect was negative in both of her eyes, and intraocular pressure was normal. As shown in Figure 1 (Fig. 1), her fundus examination revealed intraretinal and massive subretinal hemorrhage with macular involvement. In addition, vitreous hemorrhage was seen in the posterior segment examination. An MH with an approximate diameter of 600 microns was noted. The patient’s optical coherence tomography (OCT) confirmed the presence of a full-thickness MH and subretinal hyperreflective materials due to subretinal hemorrhage (Figure 1 (Fig. 1)). The fluorescein angiography revealed a focal dilation in the superotemporal first-order artery, which is compatible with RAM; and a large area of blockage, corresponding to intraretinal and subretinal hemorrhage.

**Figure 2:**
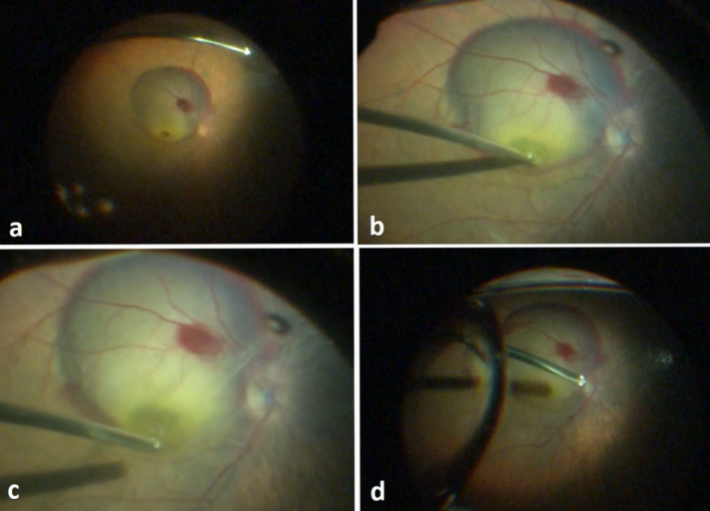
Surgical steps: a) Macular hole, submacular hemorrhage, and arterial macroaneurysm are present; b) ILM peeling; c) rtPA injection using vitrectomy probe via the macular hole, and extension of the submacular hemorrhage area after injection of rtPA; d) Fluid-air exchange at the end of the surgery

The patient has undergone a 3-port 25-gauge pars plana vitrectomy (PPV, Constellation Vision System, Alcon, Fort Worth, TX, USA). Internal limiting membrane (ILM) peeling was done. Then, 10 µg recombinant tissue plasminogen activator (Actilyse, Alteplase, Boehringer Ingelheim, France) was injected into the submacular space via the MH with a 25-gauge vitrectomy probe. The surgery was followed by fluid-air exchange and the injection of a nonexpansile concentration of sulfur hexafluoride (SF6, Eyesun Teb Co, Tehran, Iran) (Figure 2 (Fig. 2)).

**Figure 3:**
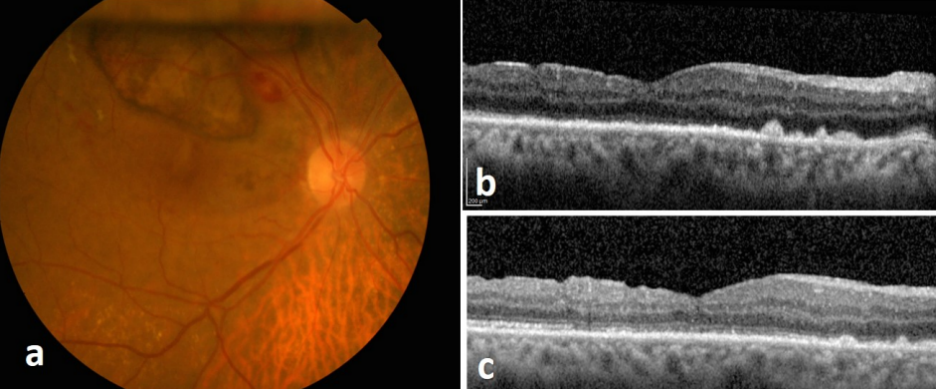
a) The clinical photograph taken 1 month after the initial presentation shows resolution of the subretinal hemorrhage with remained pigmentary changes and anatomical closure of the macular hole. b) OCT scan 1 week after surgery shows restoration of the external limiting membrane. c) OCT scan 1 month after the surgery shows that the ellipsoid zone is also recovering.

BCVA of the patient increased to 20/320 and 20/60, one and four weeks after surgery, respectively. In her fundus examination, the MH was anatomically closed and SMH was completely resolved in the macular center with some pigmentary changes. OCT images confirmed the complete anatomical closure of the MH and restoration of the outer retinal layers such as the external limiting membrane and the ellipsoid zone (Figure 3 (Fig. 3)).

## Discussion

Not as rare as previously thought, MH formation worsens the final visual outcome following the rupture of RAM [5], [8]. The exact mechanism of MH formation following macroaneurysm rupture remains unclear. Sudden highly elevated pressure in subretinal and sub-ILM space results in a gap between intraocular pressure and submacular space. Microlaceration or retinal degeneration between retinal layers can be the end result of this gap which contributes to foveal thickening and MH formation. The higher incidence of SMH in patients who have MH after ruptured RAM is in favor of this possible mechanism of MH formation [5]. In another theory proposed by Colucciello et al., vitreous hemorrhage following ruptured RAM may induce contraction at the posterior vitreous cortex, and subsequent tractional forces in vitreous may contribute to MH formation [9]. Experimental studies of SMH demonstrated severe outer retinal cell damage in the animal models [3], [4]. The possible causes of this issue were mentioned above. Therefore, early removal or displacement of SMH is necessary to achieve a satisfactory final visual outcome [8]. Furthermore, Sagara et al. showed a higher incidence of vitreous hemorrhage in patients with MH after ruptured RAM [8].

The available modalities of treatment of SMH include intravitreal gas injection with or without intravitreal rtPA injection, or PPV combined with both rtPA and gas injection. We presented a case of MH and SMH after a ruptured RAM which was treated successfully with a novel approach of PPV and rtPA injection. The ILM was removed as a standard treatment of idiopathic MH surgery. The novelty of our procedure was the use of the MH as an orifice for the injection of rtPA into the subretinal space without any further retinotomy and additional damage to the retina. However, the MH was anatomically closed as soon as one week after surgery. The final BCVA was 20/60 in our patient. Various reasons might cause the non-improvement of the final visual acuity. Among them are mechanical damage to Henle’s fibers or photoreceptors after MH formation, and the toxic effect of iron and fibrinous material for photoreceptors and the outer retina. Glatt et al. showed irreversible retinal damage within 4 hours and total loss of photoreceptors within 7 days when examining rabbit models with SMH [4].

In previous studies reporting MH formation after ruptured RAM, the final BCVA was less than 20/100 in most cases [5], [6], [7], [8], [9], [10]. This shows that despite the massive area of SMH, our patient’s visual acuity was acceptable and that she was treated successfully by this novel approach. In one study reported by Bakri et al., the authors reported a similar association between MH and SMH following ruptured RAM, and concluded that it is not necessary to remove the subretinal hemorrhage intraoperatively [10]. Uemoto et al. also reported a case of spontaneous MH closure after ruptured RAM [11]. However, considering the rapid degenerative effects of SMH on photoreceptors in animal studies, we do not suggest observational approaches for SMH spontaneous resolution.

## Conclusion

In conclusion, this case report expands our knowledge about the management of MH in combination with SMH after a ruptured RAM. This condition can have a poor visual outcome if left untreated, and prompts timely and appropriate management for a successful visual recovery of the patients. We suggest the use of a vitrectomy probe and MH for subretinal rtPA injection.

## Notes

### Competing interests

The authors declare that they have no competing interests.
